# First Principles of Surgery, Being an Outline of Inflammation and Its Effects

**Published:** 1840-10

**Authors:** 


### Art. XV.

*First Principles of Surgery, being an Outline of Inflammation and its Effects.* By George T. Morgan, a.m., formerly Lecturer on Surgery at Aberdeen.—*Edinburgh,* 1840. 8vo, pp. 780.

It gives us great pleasure to be able to repeat, in regard to the second and third parts of this treatise, which complete the work, that which we formerly stated as our opinion of the first, (Vol. IV., p. 185-8.) We regard it as one of the best guides to the study of inflammation and its effects which the surgical student can employ; characterized as it is not only by thorough knowledge of the subject, but by that wisdom which is the result, in the well-trained mind, of the acquirement of it, and which is often lamentably deficient when the stores of information are most full. The opinions of others are made by Mr. Morgan his own, not in proportion as they tally with a preconceived theory, but according as they are borne out by general experience; and the student is thus prevented from acquiring those exclusive notions in which some teachers are in the habit of training their pupils, and, on the other hand, from being perplexed by the multitude of conflicting statements which others mistake for scientific instruction.

The parts now before us are devoted to the consideration of the treatment and the (so called) terminations of inflammation. That they contain little of novelty is not to be imputed to them as a fault, for they lay claim to none; and every one conversant with the business of instruction must be aware how much more practically useful is a judicious eclecticism than the inculcation of untried doctrines, however plausible. In his treatment of acute inflammation Mr. Morgan agrees with all but the votaries of the *médecine expectante*, in considering bloodletting as the most important remedial agent we possess. We fear, however, lest his expressions on this subject may mislead his young and ardent readers into an incautious use of the lancet. In Mr. Morgan's own hands we do not doubt that is a safe as well as an efficient weapon; but we think that it ought to be more fully guarded, in the hands of the student and junior practitioner, by a knowledge of the very frequent ill effects of over depletion.

We are happy to see that Mr. Morgan does not share in the anti-mercurial mania which prevails at present in some parts of Britain. His observations on the use of this remedy in the treatment of acute inflammation we consider extremely sound. In point of importance, he places it next to bloodletting; taking care to explain, however, that it is only when employed in combination with, or subsequently to, free withdrawal of blood that its good effects can be looked for. He is quite ready to acknowledge, also, that there are some cases in which it seems to have no control over the morbid action; others in which its influence is doubtful; and others again where, either from the mode or time of its administration, it has but little effect. He has no great confidence in its employment in inflammation of the mucous membranes; and in inflammation of the substance of the brain his experience leads him to consider it as altogether useless. The following remarks on its *modus operandi* we deem well worth quotation.

"The immediate benefit to be expected from a mercurial plan of treatment is, in the meantime, a matter of speculation. The peculiar action of mercury on the system is imperfectly understood; and we are much in want of some careful and well-conducted trials, by which its application in cases of inflammation might be indubitably determined. To us its good effect seems principally to arise from its influence over the capillary circulation, in the way of altering the secretions, whether natural or morbid, in different parts of the system; and more especially of preventing or removing those depositions proceeding from continued inflammatory action in a part, and over which common evacuants appear to have no control. Were we to go more minutely into the consideration of its effects we should say that it possesses a greater power in preventing the deposition and assisting in the removal of lymph than any other species of effusion; and this more particularly in some structures, as the serous, than in others. We are of course far from wishing to assert that this is the only way in which mercury proves useful in inflammation, as its good effects are visible in cases where those changes have not yet commenced; all we mean to intimate is that its efficacy is pointed out to us at least to be in so far as we have stated. Thus, in iritis, where lymph has been thrown out on the surface of the membrane, mercury not only prevents its further effusion, but promotes the absorption of that which has been deposited. In laryngitis and croup it operates after the same manner, and checks that effusion of lymph which proves so great a barrier to respiration; although, in the latter case, from the uniform (?) extension of the disease to the bronchiæ, we must confess we have derived little satisfaction from its most liberal use, and have only been compelled to have recourse to it in the absence of a better remedy. Its influence in removing the matters deposited during local inflammation is also well illustrated in chronic enlargement of the testicle, where the continued use of mercury will alone effect a permanent cure." (p. 255.)

On this last point we may remark that Mr. Morgan scarcely gives sufficient credit, in our opinion, to the efficacy of iodine, especially when combined with mercury, in removing the products of chronic inflammation. His reference to the case of iritis, as illustrating the good effects of mercury in acute inflammation of serous membranes, seems to us extremely in point; and we have often been surprised that it has been made so little use of in the discussion. Those who have witnessed the treatment of many cases of this disease are well aware that it is not only in its syphilitic form that the good effects of mercury are evident. It is true that there are many cases that resist it; but in these its administration is usually contra-indicated by some peculiar state of the system over which it is known to have little or no control. But in the pure sthenic inflammation of the iris, brought on from exposure to cold, from external injuries, or other obvious causes, in subjects of ordinary constitutions, mercury, administered in time and with proper adjuncts, may be regarded as almost a specific. Here we have the inflamed membrane under our eyes, and the effects of the disease visible from day to day. And who is there, that has had the disease under his care, that has not seen the symptoms that were previously advancing with alarming rapidity, threatening complete loss of sight, and accompanied with intense pain, suddenly checked,—the pain diminishing, the pupil clearing and becoming regular, the flocculent masses of lymph that previously lay upon the membrane disappearing, and the patient gratefully acknowledging the improvement—on the day, nay almost the hour, that the affection of the mouth begins to show that the system is under the influence of the medicine? Now, so far as our experience goes, and it has not been small in this class of cases, we confide so much in the curative effects of mercury in uncomplicated cases of iritis, that we seldom should think it necessary to have recourse to more than topical bleeding as an adjunct. But, on the other hand, if the horror of mercurial poisoning should lead to the neglect of this remedy, little check can be put to the disease (as we have more than once seen where this plan of treatment has been attempted) save by general bloodletting, carried to an extent that cannot but be productive of permanent injury to the patient. We might speak in still stronger language of the efficacy of mercurial treatment in almost all cases of syphilitic iritis in which the constitution has not been shattered by the previous disease and by injudicious treatment of it; but that would be introducing a question foreign to our present object. We trust that we have said enough to prove that in the case in which, from local circumstances, we can most clearly trace the influence of mercury as a remedy for inflammation of membranes of the serous character, it is extremely decided. Mr. Morgan insists much, and with reason, on the necessity for rapidly bringing the system under its influence in cases in which life is threatened.

"We are perfectly convinced that much of the scepticism still lingering respecting the efficacy of mercury in inflammation arises from its not being administered in sufficient quantities......We must of course prescribe according to the seat and violence of the inflammation, and likewise the age of our patient: but if, next to bleeding, we place our hopes in the use of mercury, let it be administered with the view of bringing the system quickly under its influence; and with the remembrance before us that there is a period beyond which our best efforts will not avail."

The use of other remedies for acute inflammation is discussed with the same good sense, and with that clearness which marks the mind of one who knows how to profit by experience, whether his own or others'. The concluding remarks we consider as of peculiar value; and as we know that the plan of treatment recommended in them is often ridiculed as unphilosophical, in spite of its success, we shall quote them nearly in full.

"The treatment of acute inflammation is not to be pursued according to one steady undeviating rule, but must be modified according to many circumstances, the principal of which are the seat and duration of the disorder, the effect of previous remedies, and the powers of the system which remain. We have, in the course of the preceding pages, pointed out the plan to be adopted, when the disease is recent, in a vigorous or weakly habit; and we have now briefly to allude to the case where intense inflammation is coupled with general exhaustion........Inflammation may assume a typhoid character in consequence of active means having been neglected; or it may have done so at the commencement, and is then often united with some peculiar habit of body. In the first case it is necessary to recollect that this ultimate stage may arrive in any instance, and that the local symptoms remain, while the constitutional alter or decline. In the second, that arising from peculiarity of habit, the head is almost invariably the seat of mischief, and fatal changes will be going on in the interior of the cranium, while the vital powers are worn out, as we witness so prominently in the delirium tremens of the drunkard. Both are easily recognized by the urgency of the local symptoms, the degree of typhoid fever, the general prostration of strength, and the quick but feeble pulse.

"There is no situation, indeed, in which the surgeon can be placed where nicer discrimination is required; and on the prudent regulation of his means alone depends the chance of preserving life. It is in such cases that we are obliged apparently to break through all established rules, and adopt a line of treatment which seems at first sight to be inconsistent. If blood is taken from the arm the remaining powers are quickly extinguished; and even the operation of a purgative is attended with considerable risk. The surgeon feels himself at sea and knows not how to act. On the one hand destructive inflammation must be subdued, else fatal lesion will ensue; on the other, the system is incapable of bearing the necessary depletion, and the declining strength must be supported. Our dependence, in the first place, is placed on *local bleeding* along with *general stimuli*; and the combination of these, where there is violent inflammation in a part with little power in the system at large, is by no means inconsistent. Let us not, however, be mistaken on this subject. Where depletion, local and general, has been freely practised, and inflammation still lingers, that is not a case where the treatment we have recommended would be successful, but one which will more probably yield to counter-irritation, along with the continued use of calomel and opium or other internal means. It is where these remedies have been neglected; where inflammation has been allowed to run on without evacuants having been employed, and typhoid fever and prostration have set in; or where the disease is accompanied by these symptoms from the commencement, that the unloading of the inflamed part, along with the use of stimulants, is so advantageous. Twelve hours will, in all probability, decide the patient's fate; and we must anxiously ponder upon, and take every circumstance into account, before we determine on what course is to be pursued. The effects of the stimulants must be strictly watched, and the utmost precaution exercised that neither the depletion on the one hand, nor stimulants on the other, be carried into excess. It requires, no doubt, the greatest practical skill to adjust them; but it is in this way only that we can bring these hazardous cases to a fortunate termination." (pp. 297-9.)

Mr. Morgan is evidently referring chiefly to surgical cases; but his remarks apply equally well to fever accompanied with local excitement; and the change of practice in the treatment of this disease—the administration of stimulants where they would have been formerly considered as completely contra-indicated—has been productive of the happiest consequences.

The treatment of chronic inflammation is next described with the same good sense: and from this the author goes on to adhesion. The chapter on this subject we are disposed to regard as the least modern of any part of the work. Mr. Morgan is a zealous upholder of the Hunterian doctrine of *adhesive inflammation;* and not to mention Dr. Macartney, whose work was published, we believe, some months before the second part of Mr. Morgan's, the opinions of some other authors than John Bell should have been noticed on the other side of the question. Under the same title we find, what we should scarcely have expected, the whole subject of hemorrhage included. The two are certainly closely connected in practice, but the data upon which the principles of their treatment are founded are sufficiently different.

The third part commences with suppuration, which is very ably treated and explained according to the knowledge supplied by the most recent observations. He zealously upholds, in opposition to Hunter, the doctrine that pus is always to be regarded as a product of inflammation; and here we are more disposed to agree with him than in regard to organizable lymph. Under this head hectic fever is ably described; although, as Mr. Morgan justly remarks, "it is a sympathetic condition of the system, not to be viewed as alone dependent on excessive discharges of purulent matter, but as arising likewise from local irritation, where little or no pus may be secreted. Its immediate exciting cause is undeniably some local disease, often a great and incurable one, where there is no power of reparation in the part itself, no assistance to be derived from nature or art, and which the constitution vainly endeavours to hold out against and shake off." (p. 545.) It can scarcely be denied, however, that the peculiar form of irritative fever which is termed *hectic* occurs in that state of the system in which there is a tendency to the formation of pus if not an actual elaboration of it. We quite agree with Mr. Morgan that the opinion entertained by some of hectic fever being caused by the admixture of purulent matter with the mass of the blood, has no real foundation. But just as we observe a peculiar accumulation of fibrine in the blood, when there is a tendency to the effusion of coagulable lymph, so can we readily conceive that there may be some other morbid change in it favorable to the secretion of pus, and producing hectic fever, as the former does the ordinary constitutional irritation.

Ulceration is next discussed, and with considerable minuteness. The author labours to impress on his readers the necessity of making themselves acquainted with the characters of different kinds of ulcers, as their chief guide in their treatment, both general and local.

"Our skill in this branch of our art is manifested by the ability of selecting the proper remedies for each kind of sore coming under our notice, and varying these from time to time as may be required. Nothing is more striking than the rapid change which ensues, whether for better or worse, according as the measures which are employed are suitable or otherwise. In no other external disease are such a variety and change of applications necessary; and hence the extent to which the surgeon must draw upon his own ingenuity and information. As has justly been remarked, it is in the management of ill-conditioned sores and ulcers, whether of a simple or specific nature, that the daily and ordinary duties of the surgeon consist; and it is by his success in treating them that his skill in practice will be judged of by his employers. Out of twenty surgeons who can perform all the operations in surgery with the requisite dexterity you will not probably find more than one who can treat properly the ill-conditioned sores or ulcers in which even the wounds necessarily inflicted by operations sometimes terminate. We would fain hope that this ignorance is not now so prevalent." (p. 610.)

In this hope we cordially unite; for the condition of the legs of her Majesty's subjects must indeed be pitiable if so small a proportion of surgeons as Mr. Morgan hints at should be competent to give them effectual assistance. In his principles of treatment Mr. Morgan adopts a judicious medium between the exclusively *local* treatment which some rely on, and the exclusively *constitutional* which in the opinion of others is sufficiently effectual to render topical remedies of little avail, and minute shades of discrimination between the varieties of ulcers worse than useless. Mr. Morgan has not found the application of blisters over and around callous ulcers, as recommended by Mr. Syme, as efficacious as it appears to have proved in that gentleman's hands. This is the fate of all remedies. The proposer is invariably more successful than any of his followers. Sometimes this is due (we regret to be obliged to believe) to an intentional magnifying on his part of the success of his treatment; more often, we trust, this is done with complete unconsciousness; and in many cases—very probably in the one before us—it arises from the acquirement by the originator of a perception of the cases in which his remedy is likely to prove beneficial, which he cannot communicate by a verbal description. We have ourselves witnessed all the good effects of this plan which Mr. Syme attributes to it; and yet M. Morgan speaks of it as producing excessive pain, and occasionally causing the most violent irritation and inflammation of the leg. We suspect that these must have been cases in which Mr. Syme would not have employed it.

The remainder of the volume is devoted to mortification; which subject is treated in the same full but lucid manner with those we have already noticed with approbation; especial pains being bestowed on the practical portion, upon which the bearing of the theoretical is, we regret to say, not yet very decided. It is evident that, keeping in view his great object of supplying the student and junior practitioner with a *practical* guide to this most important branch of the principles of surgery, he has here as elsewhere avoided lengthy discussions on controverted points, however abstractedly interesting; and in the wisdom of this course we fully concur with our author.

---

